# Comparison of the vertical accuracy of satellite-based correction service and the PPK GNSS method for obtaining sensor positions on a multibeam bathymetric survey

**DOI:** 10.1038/s41598-024-61591-5

**Published:** 2024-05-15

**Authors:** Walmor Pereira de Andrade Neto, Igor da Silva Rocha Paz, Raquel Aparecida Abrahão Costa e Oliveira, Maurício Carvalho Mathias De Paulo

**Affiliations:** Military Institute of Engineering, Rio de Janeiro, Brazil

**Keywords:** Bathymetric survey, Satellite-based augmentation system, Postprocessed kinematic, Hydrographic survey, GNSS, GPS, Ocean sciences, Engineering

## Abstract

The MarineStar Global Navigation Satellite System (GNSS) augmentation services are widely used in bathymetric surveys to obtain the sensor position. The postprocessed kinematic (PPK) GNSS method can be used to obtain more accurate positions. Therefore, the present work aims to verify the vertical final accuracies and uncertainties of exclusive order multibeam hydrographic surveys using the Global Navigation Satellite System (GNSS) as a horizontal positioning tool through methods such MarineStar services and PPK relative positioning, with comparisons between the two methods based on the results of the hydrographic base created. We produced two surfaces with each method, representing the minimum and maximum depths of the bathymetry point cloud. A statistical analysis of the differences between the depth surfaces obtained with the two methods was carried out. When comparing the two surfaces using the PPK method, the difference was less than 10 cm at 95.1% of the points. When comparing the two surfaces to those of the MarineStar, the difference between the maximum and minimum depths was less than 10 cm at 93.1% of the points. The results suggest that using the same survey data, the products obtained with the PPK method would comply with the hydrographic standards, while the MarineStar products would not.

## Introduction

Along with rail transportation, water transportation has key importance in regard to extensive displacement and a substantial amount of cargo or passengers, as well as its low logistical cost for handling and minimal influence on environmental pollution. With great importance in the Brazilian context, one of the biases of waterway transport, inland navigation, which occurs mainly in rivers, is worth highlighting, with Brazil having an extensive area to be explored^1^.

To enable the operation of all waterways, it is necessary to manage the charts of all waterway infrastructures within the specific parameters provided by the control agencies. In that context, one of the engineering techniques used is bathymetry^2^. For the generation of spatial information by means of bathymetry, qualified professionals registered with the Directorate of Hydrography and Navigation develop Hydrographic Survey, with the Brazilian Navy responsible for making and updating all the Brazilian hydrographic charts^3^.

Through the Brazilian Navy, norms and procedures were established for the authorization and control of the Hydrographic Surveys carried out in Brazilian Jurisdictional Waters by a body or entity not belonging to the Brazilian Navy itself. Hydrographic surveys classified as exclusive order must fully comply with the specifications of S-44^4^, so that they can be used to update nautical documents.

**Figure 1:**
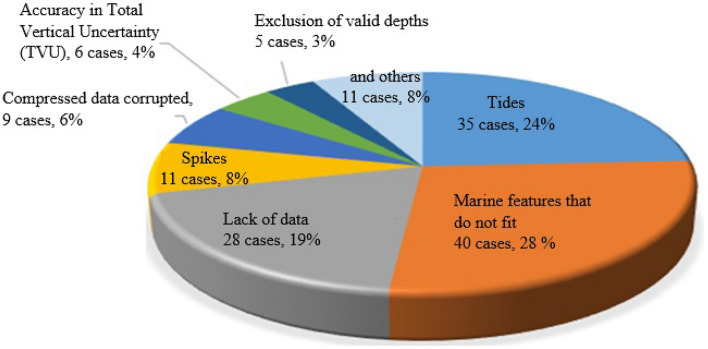
The 145 cases analyzed from 2014 to 2018. *Source* Brazilian Navy, Directorate of Hydrography and Navigation, Navy Hydrography Center.

In a technical visit by the Directorate of Hydrography and Navigation, it was possible to obtain results referring to 145 cases of all hydrographic surveys of exclusive order, authorized and not used between 2014 and 2018. The following inconsistency values were obtained: marine features that do not fit, 40 cases representing 28% of all inconsistencies; tides, 35 cases, 24%; lack of data, 28 cases, 19%; spikes, 11 cases, 8%; compressed data corrupted, 9 cases, 6%; accuracy in total vertical uncertainty (TVU), 6 cases, 4%; exclusion of valid depths, 5 cases, 3%; and others, 11 cases, 8%, as shown in Fig. 1^5^.

**Figure 2:**
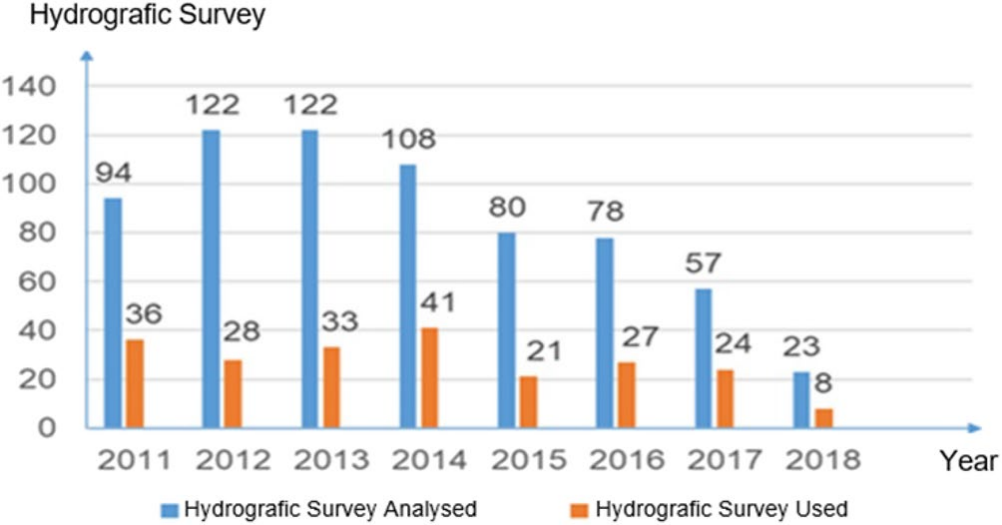
Number of hydrographic surveys analyzed and used by the Brazilian Navy between 2011 and 2018. *Source*^5^.

By analysing historical data, it is possible to verify that a large number of hydrographic surveys authorized by the Brazilian Navy were not used by the entity for the preparation or updating of new hydrographic charts. Figure 2 shows the number of hydrographic surveys of the exclusive order analysed and used by the Brazilian Navy over 8 years of historical data (2011–2018). It is possible to verify that between 2011 and 2018, 684 surveys were analysed, and 218 were used, representing 32% of the analysed surveys.

Aiming to develop an alternative that could collaborate to reduce the nonuse of hydrographic surveys, this research compares two positioning methods by GNSS to improve the precision and final bathymetric accuracy. The Satellite-Based Correction Service (MarineStar), regularly used in hydrographic work^5^, and the PPK method, established for some applications in terrestrial cartography, were compared. The results were compared with the hydrographic survey technical requirements of the Brazilian Navy Exclusive Order to verify whether the difference in precision provided by the two methods would produce hydrographic bases with different chances of use, mainly regarding the marine features that do not fit, the accuracy in total vertical uncertainty (TVU) and the accuracy in total horizontal uncertainty (THU).

Data from a multibeam hydrographic survey developed in Porto do Pecém, CE, were used. The survey was carried out through geodetic and bathymetric studies in the area of interest, referred to as the hydrographic datum, after the construction of berth 9 and the new access bridge to the multiple use terminal (TMULT per its Portuguese acronym).

## Literature review

According to Dautermann et al.^6^, the GNSS is divided into three categories: global systems, regional systems, and augmentation systems. In this study, issues related to the MarineStar and the GNSS PPK relative method will be addressed.

Many systems are designed to provide more data to improve the GNSS positioning. For example, the Satellite Based Augmentation Systems (SBAS) provide data to users using a signal of the same frequency as the L1 signal of the GPS (1575.42 MHz) but in a different format^7^. SBASs can transmit correction messages and additional guarantee parameters to increase the reliability of GNSS users^8^. For instance, satellite orbit and clock corrections, ionospheric corrections and estimated errors are associated with the ionosphere^9^. There are commercial systems, such as MarineStar, that uses satellite communication to provide corrections to receivers. MarineStar also provides the same RTCM messages through the internet over the NTRIP protocol^10^.

The MarineStar commercial system can provide decimeter precision using DGNSS, dual-frequency receivers and up to 4 satellite constellations. The reception can be via a dedicated small omnidirectional antenna or an existing compatible Inmarsat system. These options allow corrections to be obtained from MarineStar without the need for positioning related to a base station^10^.

For the configuration of the relative positioning method, one or more fixed stations (base stations) need to be materialized with known coordinates for reference. The mathematical model requires that both receivers can track the same satellites simultaneously. With short baselines smaller than 10 km and “calm” ionospheric conditions, the atmospheric and orbital errors of satellites become practically null, triggering solutions for these ambiguities^11^. For baselines above 10 km, dual-frequency equipment is recommended for reducing ionospheric effects^12^. The tropospheric residual effects associated with the application of a certain model are estimated through additional parameters that are calculated by certain programs^13^. When searching for superior precision, the ephemeris and the error of the satellite clock must be acquired from external sources, identical to precise point positioning (PPP)^14^.

Several studies have verified differences in accuracy in bathymetric surveys due to the vessel positioning method. McHugh et al.^15^ compared the accuracy of the bathymetry products obtained using four different methods: precise point positioning (PPP), real time networks (RTN), real time kinematic (RTK) and PPK, using virtual reference stations (VRS). The results showed that only PPP and PPK were reliable to meet the IHO standards. Nakao and Krueger^16^ evaluated the differences in vessel trajectories via a bathymetric survey performed by three different methods: PPP, differential positioning and PPK. The research indicated that the PPK procedure had the best accuracy. Another study reported worse results for the PPK than for the PPP in the case of a survey 300 km from the coast^17^. Elsobeiey et al.^18^ compared the bathymetric accuracy using two methods: a satellite-based correction service, Trimble PP-RTX, and RTK with VRS. Unlike McHugh, both methods achieved products compliant to IHO standards. Our study presents an evaluation similar to McHugh et al.^15^ and Elsobeiey et al.^18^, but we compare the depths on the products obtained through PPK, with a survey station, and the MarineStar augmentation service. Despite the broad scientific approach to some GNSS methods applied to hydrography, none have compared MarineStar and PPK for multibeam bathymetry in Special Surveys.

## Methodology

We performed a multibeam bathymetric survey and processed the same data using two different methods, changing only the GNSS position of the vessel. In this way, the other error sources inherent to the bathymetric survey were repeated in both methods. The bathymetric survey met all the S-44 requirements for a hydrographic survey of the exclusive order. For this purpose, it is necessary to cover 200% of the entire region to be mapped; that is, at least two complete surveys of the entire area of interest are needed. To take advantage of the hydrographic survey, the Brazilian Navy performs an evaluation of the surfaces generated for all defined cross-sections. These cross-sections should differ by less than ten centimetres in up to 95% of all cases.

**Figure 3:**
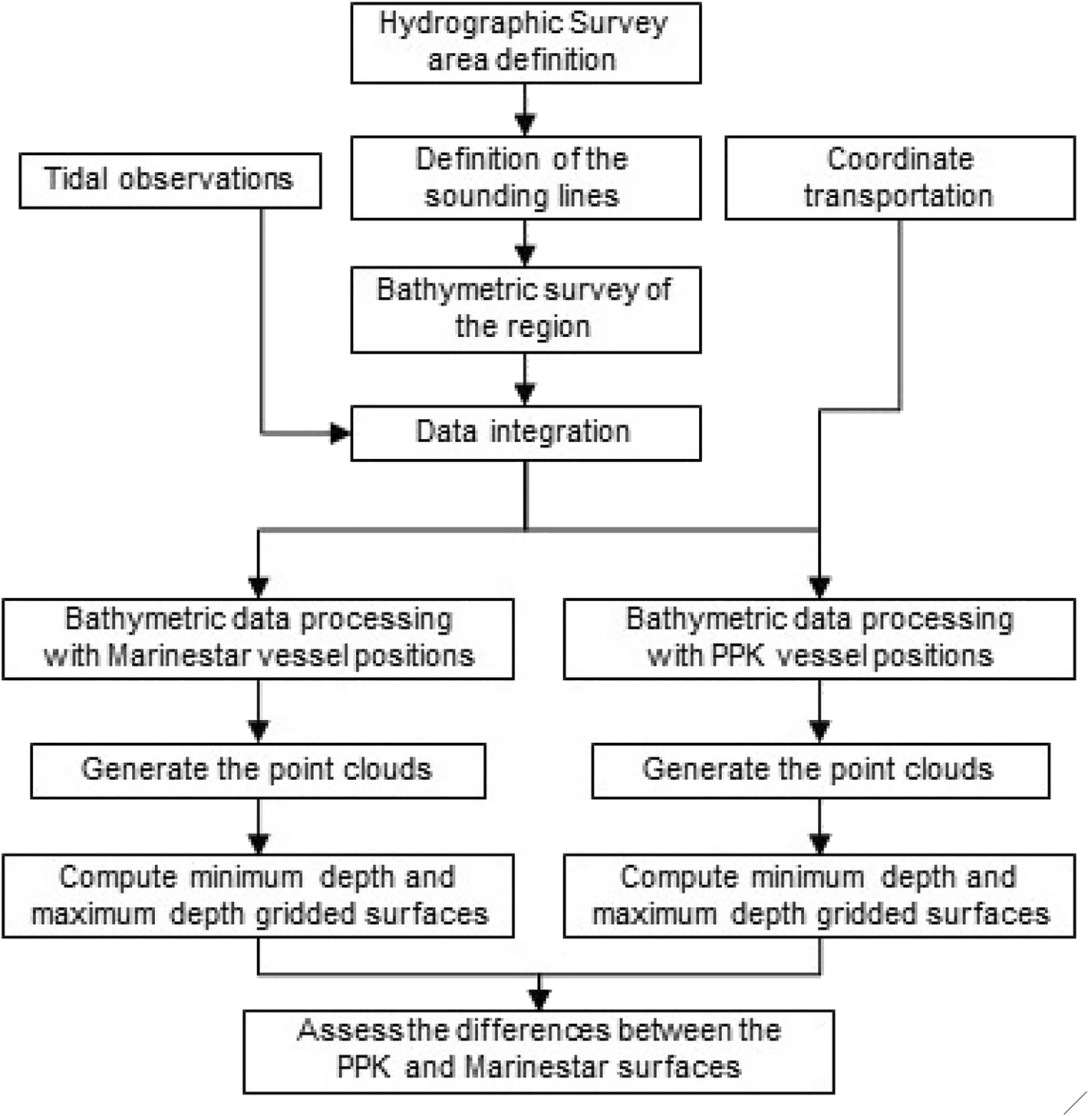
Study methodology flowchart.

During the bathymetric surveys, in the navigation phase, the vessel was oriented using MarineStar. The MarineStar positions were obtained in real time using single frequency GNSS (GPS + GLONASS) pseudorange and carrier phase observations. The GNSS receiver also collected dual frequency data for the PPK processing. The data was only recorded when the ambiguity resolution achieved a fixed solution. After the two surveys, two types of positioning were provided for the vessel, one of which maintained MarineStar positioning with the development of all hydrographic processing and the generation of the final bathymetric profile (coordinates “XYZ”) and later modified the GNSS horizontal position data by horizontal positioning in PPK mode for the same surveys, with the development of new bathymetric processing and the generation of new “XYZ” coordinates. Therefore, two positioning methods were used to carry out the bathymetric processing and final editing of all the data in both hydrographic surveys: Method 1—MarineStar) and Method 2—PPK. Method 1 uses absolute positioning MarineStar, while Method 2 addresses PPK. A reference station was installed in the vicinity of the hydrographic survey area as a basis for the relative method. Figure 3 presents a flowchart with all the steps developed in the hydrographic survey.

### Hydrographic survey region: Port of Pecém

**Figure 4:**
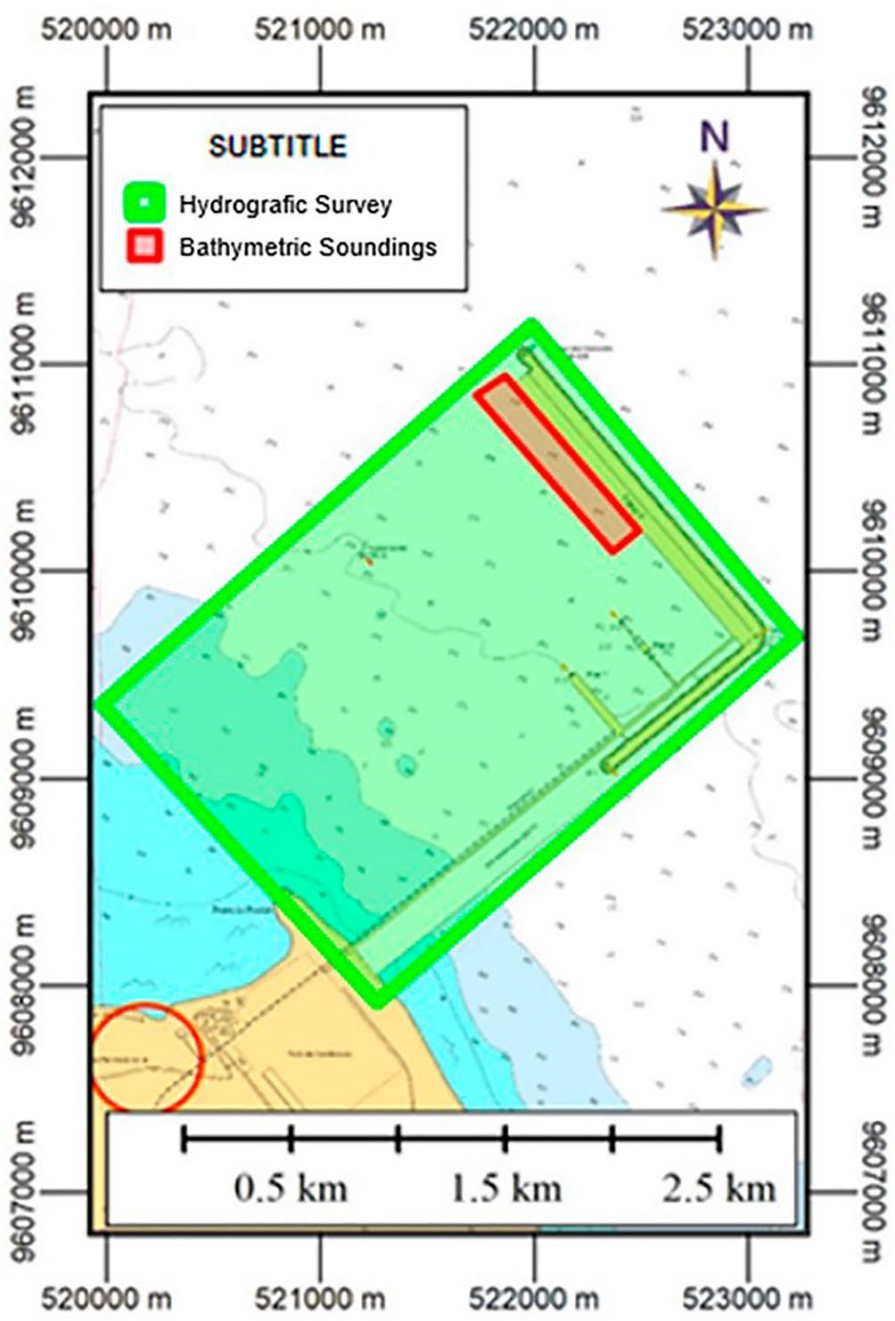
Excerpt from the nautical chart 710, with the representation of the areas where the hydrographic survey activities were carried out (green) and the bathymetric sounding (red). *Source* Adapted from the nautical chart 710.

The study area of this work covers part of the area of the Port of Pecém, indicated by the green quadrilateral in Fig. 4. The Port of Pecém is located in the District of Pecém in the city of São Gonçalo do Amarante in the state of Ceará (CE) in Brazil, 50 km from the state capital (Fortaleza/CE) and with an estimated population of 49,903 people according to the Brazilian Institute of Geography and Statistics^19^. This port has outstanding relevance in the national scenario of cargo transportation. The hydrographic survey that will be the subject of scientific study was carried out within the scope of authorization 318/2019 of the Navy Hydrography Center. Bathymetric soundings were carried out at berths 7, 8 and 9 and are indicated by the red square in the same figure.

### Coordinate transportation

It was established as a base station for geodesy activities with reference to level No. 4 (RN 04) of the tidal station of reference No. 30337—Pecém Port Terminal (F41-705-001/00) for the georeferencing of the cargo terminal pier line (TMULT) and of the new bridge to access the terminal, consequently enabling the cartographic update.

The static tracking method was used, where the high-precision coordinates of the Normative Resolution 04 (RN 04) were obtained by coordinate transporting from two stations of the Brazilian Network for Continuous Monitoring (RBMC per its Portuguese acronym) closest to the area of interest (CEFT and CEEU). The RN 04 (base station) was used as a reference for the entire hydrographic survey. Table 1 presents the transverse universal projection system of Mercator (UTM) and the geodetic coordinates.

**Table 1:** Coordinates of the tracked points.

Coordinates of the tracked points					
Name	UTM		Geodetic		Ellipsoidal height (h)*
	North (m)	East (m)	Latitude	Longitude	
CEFT	9,589,820.632	558,529.724	3° 42ʹ 38.92218ʹʹ S	038° 28ʹ 22.50428ʹʹ W	4.911
CEEU	9,571,386.164	563,778.662	3° 52ʹ 39.17574ʹʹ S	038° 25ʹ 31.94614ʹʹ W	21.749
RN 04	9,609,166.358	522,441.294	3° 32ʹ 09.33880ʹʹ S	038° 47ʹ 52.59874ʹʹ W	− 3.074

*The ellipsoidal heights were obtained with the RBMC itself as reference.

In geodesy activities for the transport of coordinates, two Trimble 5700 GNSS receivers of dual frequency (L1/L2) were used, with horizontal and vertical precision (in the static method): 0.5 mm + 0.5 ppm for planimetry and 0.5 mm + 1 ppm for altimetry. The geocentric reference system used was the Geocentric Reference System of South America in 2000 (SIRGAS 2000, per its Portuguese acronym), zone 24 S, central meridian 39° W and ZULU time zone (UTC).

### Tidal observations

In accordance with the Brazilian Navy provision of tide chart observations at the extremes of the sounding region, with the purpose of verifying the need to carry out tidal zoning, it was verified that there were no differences in phase, amplitude or tidal form between the two stations located within the limits. Thus, in the hydrographic survey, aiming to establish a tide gauge station in the drilling region, to reduce the bathymetries, which were carried out from berths 7, 8 and 9 of the TMULT, which totaled an area approximately 700 m long and 200 m wide, berth 9 station was occupied, whose tidal elements were obtained by cross-harmonic analysis (AHC per its Portuguese acronym), with the harmonic constants of station 30337.

### Hydrographic survey area

In this section, we describe further details of the survey in which data were simultaneously collected for both survey methods. Both methods use the same sounding lines and equipment, resulting in the same GNSS observations being made at the rover receiver. The processing software and external data used were different for each processing method.

#### Definition of the sounding lines

The regular bathymetric soundings of the project area were carried out on the 29th and 30th of August 2019. To establish a total coverage of the bottom and 100% overlap, a 10-m grid of regular sounding lines was used for verification.

#### Configuration and setting of the bathymetric system

The bathymetric soundings were carried out with the vessel Penedo II (AB 24.00, draft: 0.95 m light and 1.42 m loaded), with the multibeam system installed on a rod on the side.

Bathymetric data were acquired using a multibeam echo sounder (MBES), R2Sonic 2024 model, operated at a frequency of 300 kHz. The equipment was installed at the edge of the sounding vessel on a rod fixed to its structure.

#### Bathymetric survey

The bathymetric soundings were carried out with a multibeam system (Multi Beam Echo Sounder—MBES) from the R2Sonic mod. 2024, installed on the edge of the sounding vessel on a specific rod. Thus, for the correct acquisition of bathymetric data, the precise determination of the offsets was fundamental by carrying out dimensional control. Data processing was carried out in two steps to compare the two intended methods: Method 1—MarineStar, Method 2—PPK.

#### Bathymetric data processing: Method 1—MarineStar

This method is pertinent to the absolute tracking mode, with no need for a base receiver to perform mobile receiver corrections. All protocols for establishing multibeam bathymetry were developed, with the horizontal positional delivery fluctuating in centimeter precision values and approximately 20 cm of uncertainty. After the development of the entire survey through this method, all the steps for processing and editing the data were carried out, with accurate values relevant to this method being delivered to the system.

#### Bathymetric data processing: Method 2—PPK

To obtain horizontal positional data via this method, a base station (RN 04) was used as a correction, which was classified in relative tracking mode. In this mode, the base station is turned on before the work starts and turned off after the conclusion so that all corrections can be made by postprocessing. During navigation, MarineStar was used to guide the vessel while collecting bathymetric data.

After completing the entire survey, the vessel’s positioning data were processed, using the base station as a reference. The vessel’s postprocessed coordinates obtained by PPK were inserted in place of those obtained by MarineStar. The bathymetric processing was repeated with these new coordinates, creating new hydrographic data based on the same survey data used in Method 1.

## Results and discussion

Initially, checks were made of the geodetic framework that supported all the work to visualize the disparity in horizontal accuracy to the MarineStar and PPK. It was verified through the analysis of the final coordinates for the two methods (DGNSS and PPK), after eight hours of occupation that the positional inaccuracy for MarineStar was between 10 and 20 cm throughout the day, while that for the PPK was less than 1 cm.

To visualize the horizontal precisions during the survey as a function of the two tracking methods, the accuracy corresponding to each type of survey was extracted from the manager program for the two positioning methods. A cross section of the surveyed area was used to verify the accuracy of the horizontal position data at the time of the survey.

**Figure 5:**
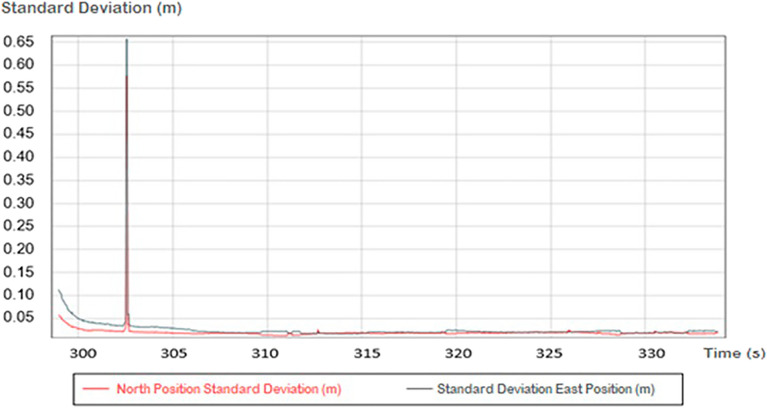
Images representing the typical deviations to the North and East for several positions in a given time range. The data were extracted from Trimble’s PosPac MMS program.

Figure 5 shows the SDs to the north and east of the vessel position obtained using the differential L-band (MarineStar) G2 (Method 1). It is observed that, in the time range illustrated, the horizontal precision reached is decimetric. In most of the illustrated period, the typical deviations for both the North and East were less than 10 cm in most cases for this section.

Both before 300 s and between 300 and 305 s, it is possible to observe peaks with higher standard deviation values, showing that in unfavorable situations, the accuracy of the system is extremely compromised, reaching values on the order of 1 m, as can be seen between 300 and 305 s. In this example, the number of satellites available in this epoch were significantly lower (under 7). Other issues related to real time GNSS could provide a similar effect to MarineStar positioning.

**Figure 6:**
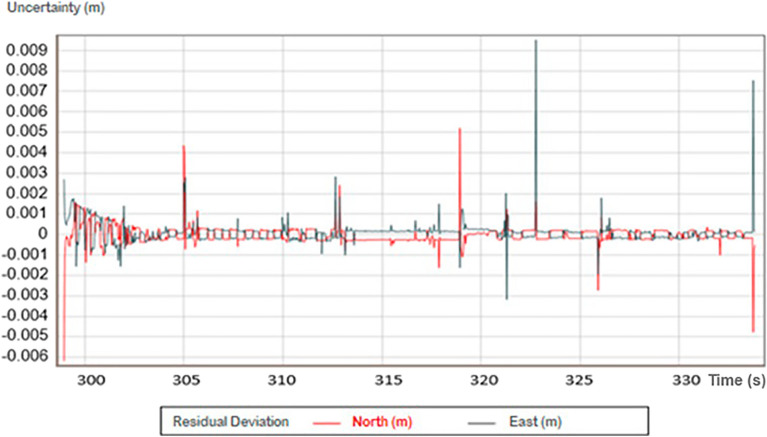
Image representing the positioning uncertainty for a given time range. The data were extracted from Trimble’s PosPac MMS program.

When processing the positions using Method 2 (PPK), the horizontal accuracy improved markedly, down to the millimeter scale. Figure 6 shows the same data presented in Fig. 5 processed for the same time range, showing the result for each instant of the cross-section. The accuracy achieved with PPK reached millimetric values, being less than 1 cm in 100% of the patients. The difference between Figs. 5 and 6 suggests that the PPK method achieved better accuracy on epochs with a low number of satellites.

Two multibeam bathymetric surveys were carried out over the entire area of berths 7, 8 and 9, making it possible to overlap 100% of the two surfaces generated from each positioning method, resulting in a coverage of 200%.

After the two hydrographic surveys were completed with the coordinates provided by each GNSS tracking method, bathymetric processing, editing and generation of the final hydrographic base were carried out with “XYZ” coordinates. Therefore, for each method, the same surveys generated two surfaces: one of maximums and one of minimums. The maximum surface area represents the greatest depth at each coordinate. Similarly, the minimum surface area represents the lowest mapped depth. The survey was planned with 10 m between the survey lines with 80° of swath angle, obtaining a planned lateral overlap of more than 60%. The points were collected with a nominal density of 1 point/cm^2^. The generated surface was represented by a grid of 515,981 “XYZ” points, with a spacing of 0.5 m as a result of a regular grid.

**Figure 7:**
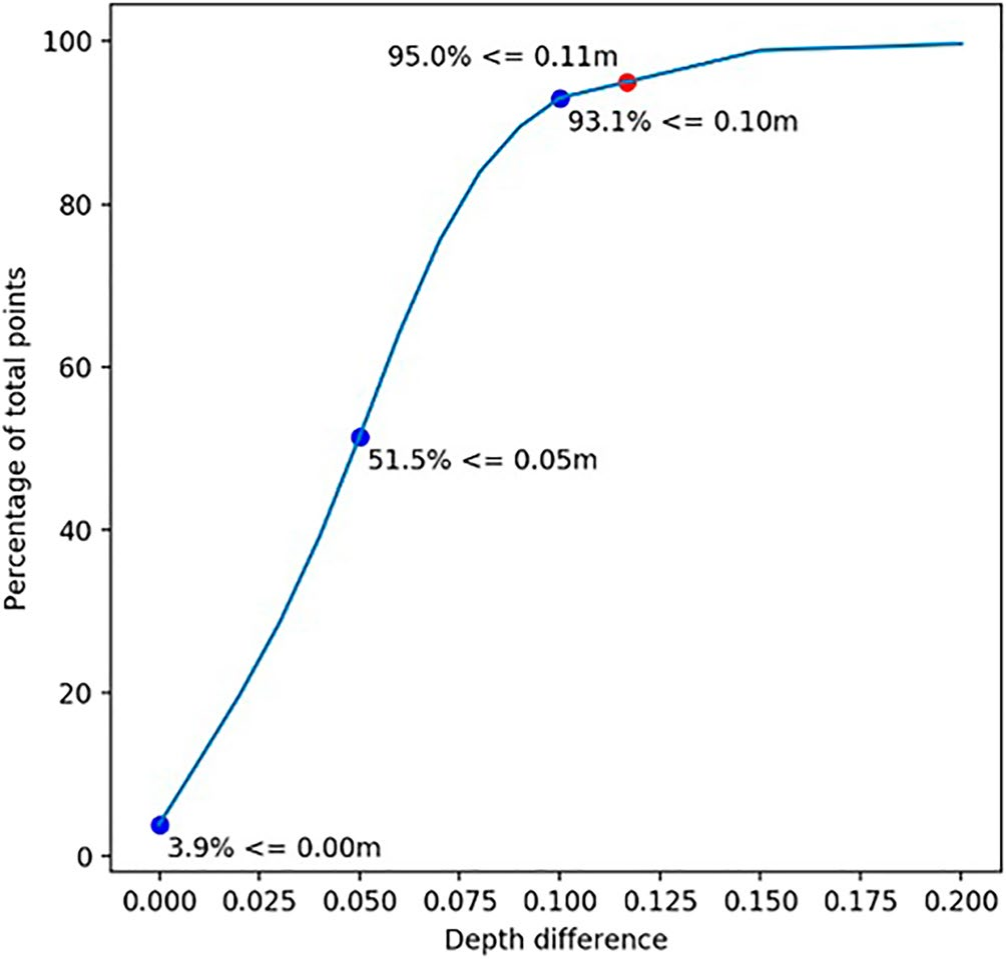
Images representing the percentage of points by precision.

Comparing the two bathymetric surfaces generated from Method 1, it was found that in 93.1% of the cases, the points of the minimum and maximum surfaces had a vertical difference lower than or equal to 10 cm. This result could make it unfeasible for the Brazilian Navy to use this hydrographic survey to meet the Exclusive Order, which requires at least 95% of each cross-section. Figure 7 depicts the percentage of points as a function of the difference in depth between the two surfaces generated. As shown in Fig. 7, in 95% of the cases, the points had a vertical difference lower than or equal to 11 cm.

**Figure 8:**
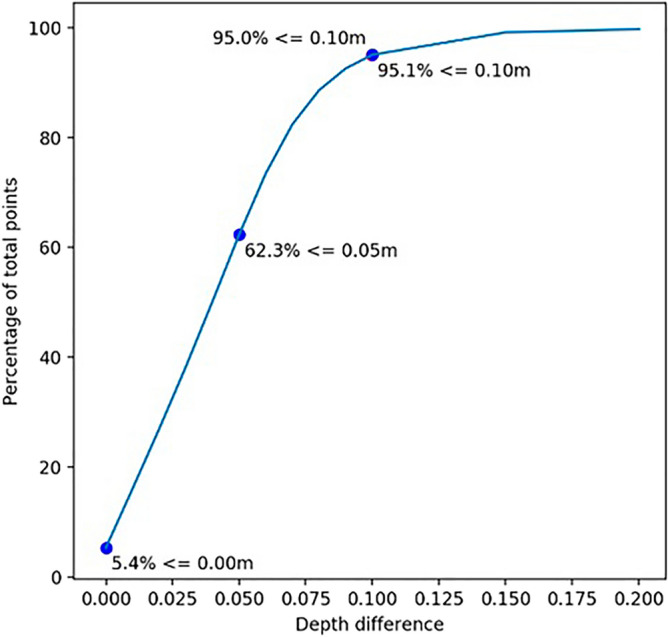
Images representing the percentage of points by precision.

A comparison of the two bathymetric surfaces generated via Method 2 revealed that in 95.1% of the patients, the points had a vertical difference lower than or equal to 10 cm. This result tends to be acceptable for the specification imposed by the Brazilian Navy for the Exclusive Order, which specifies that for all cross-sections, there must be a difference between the maximum and minimum depths lower than or equal to 10 cm in at least 95% of the cases. Figure 8 depicts the percentage of points as a result of the difference in depth between the two surfaces generated.

**Figure 9:**
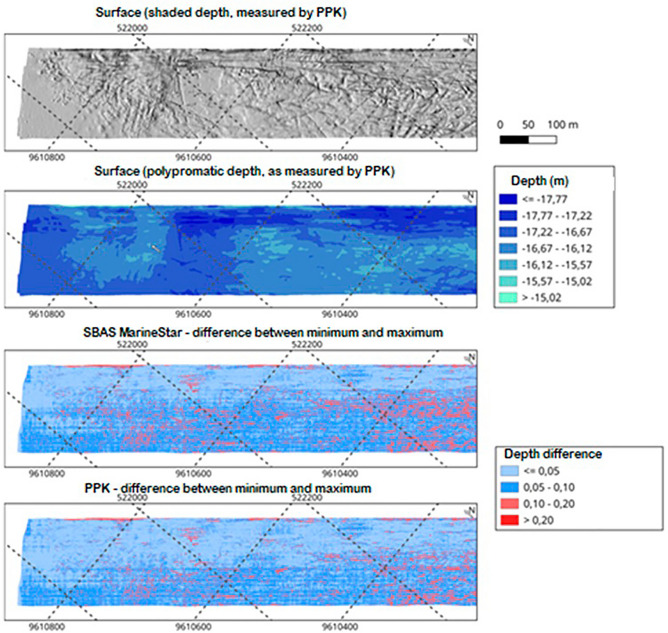
Differences between the minimum and maximum surfaces using MarineStar and PPK (at the bottom). The surface maps (on the top) show the roughness and depth of the survey area.

Figure 9 shows an illustration of the four surfaces. The first map represents, in gray and shading levels, the surface depths of the minima of the hydrographic survey delivered and used by the Brazilian Navy to update the local Nautical Chart produced by Method 2. The Brazilian Navy uses the surface that has the lowest depth and is conservative to preserve the safety of navigation. The second map represents the same depths on a blue scale, without shading, to visualize the deepest regions of the study area. The other maps show the depth differences between the two surfaces (minimum and maximum) produced with each positioning methodology, Method 1 and Method 2. The shades of red represent regions that do not meet the Brazilian Navy requirements, that is, that surpass 10 cm of disparity between the two surfaces.

It is possible to visualize monochromatic map areas at more homogeneous depths and others at more heterogeneous depths. Note that in the PPK image, lighter blue tones prevail, which highlights more precise regions. The MarineStar surface shows a higher number of points in light and dark red tones, representing higher inaccuracy than that of the PPK model. The more heterogeneous a given area is, the more important the horizontal positioning accuracy is.

**Figure 10:**
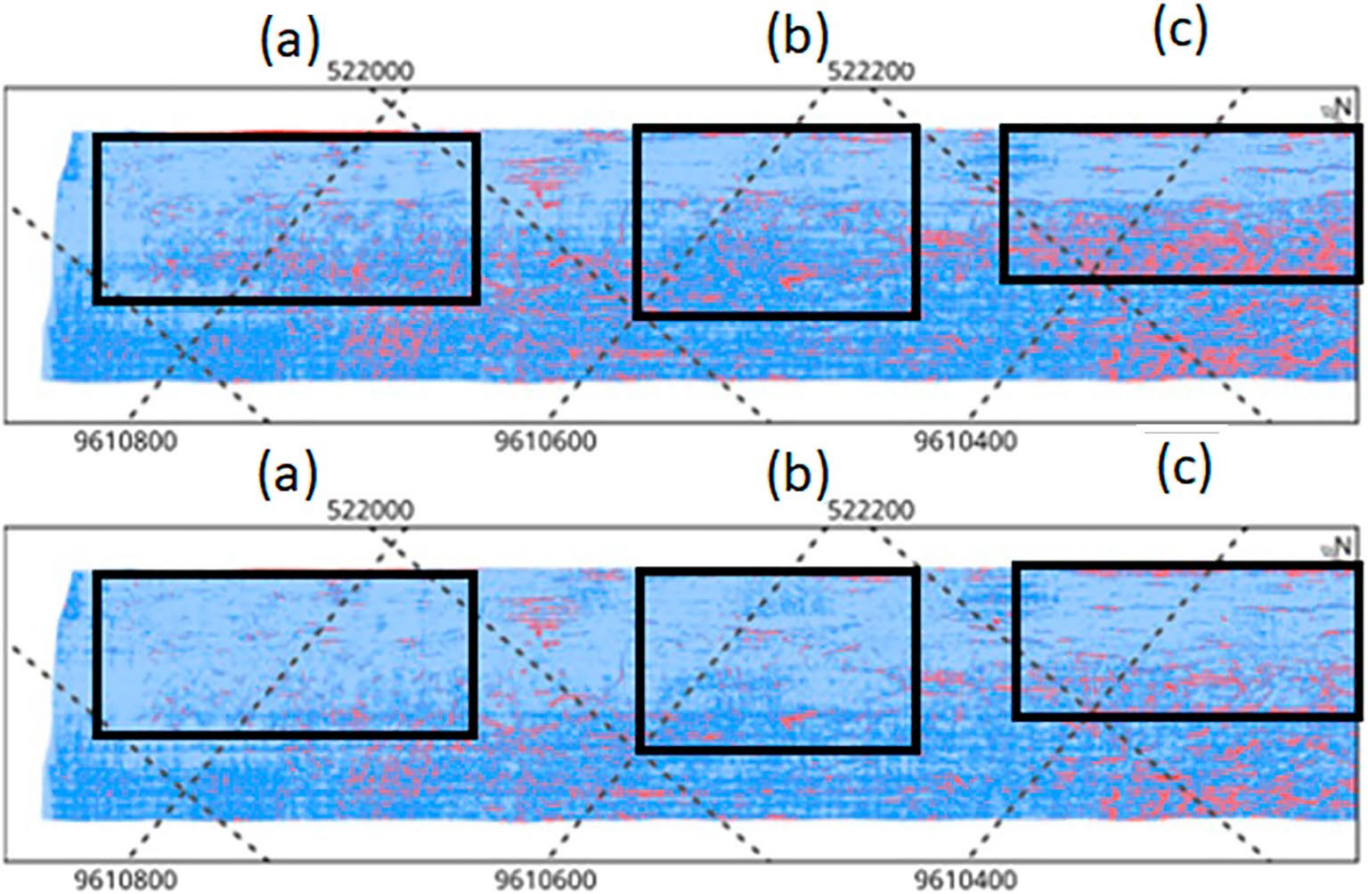
Visual difference in precision for the common areas (**a–c**) between the images generated by MarineStar and PPK.

Next, Fig. 10 shows three areas (a, b and c) common to images generated for the MarineStar and PPK surveys, where it is possible to visually observe the differences in precision due to the disparity of the blue and red tones common in both images.

Through visualization, the difference in precision is distinctly evident when we analyze the sampling in areas a, b and c with the respective interpretations.

**Figure 11:**
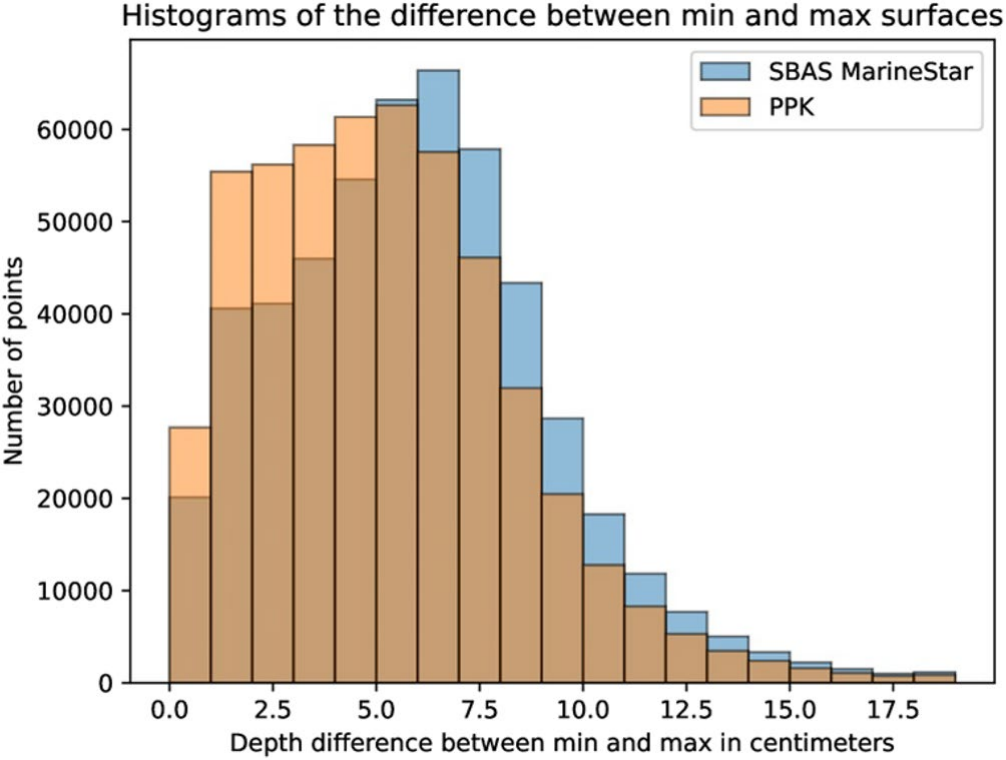
Histogram of the number of points by precision.

To visualize the influence of the horizontal positioning precision on the vertical coordinates, a chart of noncumulative values (histogram) was generated. Figure 11 illustrates the difference in the depths of the pairs of models for each method according to the number of points. The brown shading represents the number of points for each accuracy range that is common to both methods, MarineStar and PPK. The beige color represents the number of points uniquely pertinent to the PPK method. The blue color represents the number of points uniquely pertinent to the MarineStar method.

A greater number of PPK points in the chart located within the range of 0 to 5 cm can be verified. There was a greater incidence of MarineStar points beginning at 6 cm. The surfaces obtained from each method were developed from the same surveys. The better accuracy of the vessel position obtained by method 2 (PPK) contributes to improving the accuracy of the resulting hydrographic base.

**Figure 12:**
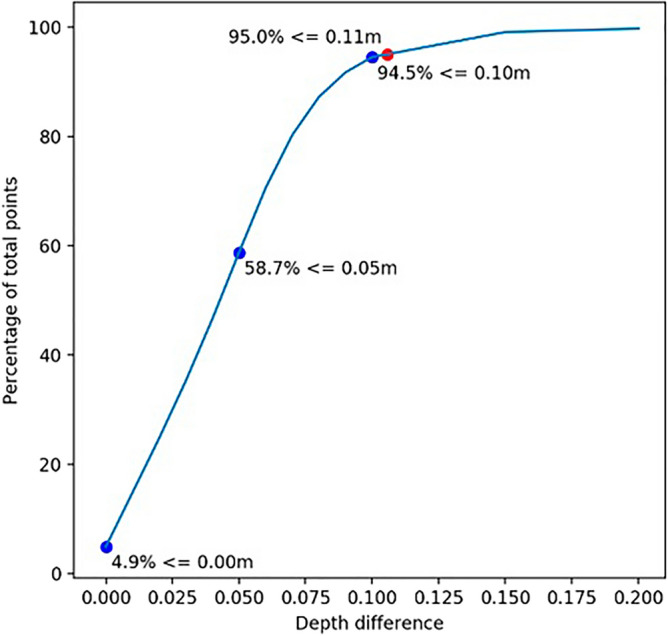
Images representing the percentage of points by precision.

A surface representing the difference between the minimum surfaces derived from each positioning method was also produced. Figure 12 depicts the percentage of points as a function of the difference in depth between the two generated bathymetric surfaces. It is possible to notice in the figure that in 94.5% of the patients, the points had a vertical difference lower than or equal to 10 cm.

In the specific case shown in Fig. 12, the minimum hydrographic bases (shallowest) were compared; that is, would be the result delivered to the Brazilian Navy according to each positioning method.

**Figure 13:**
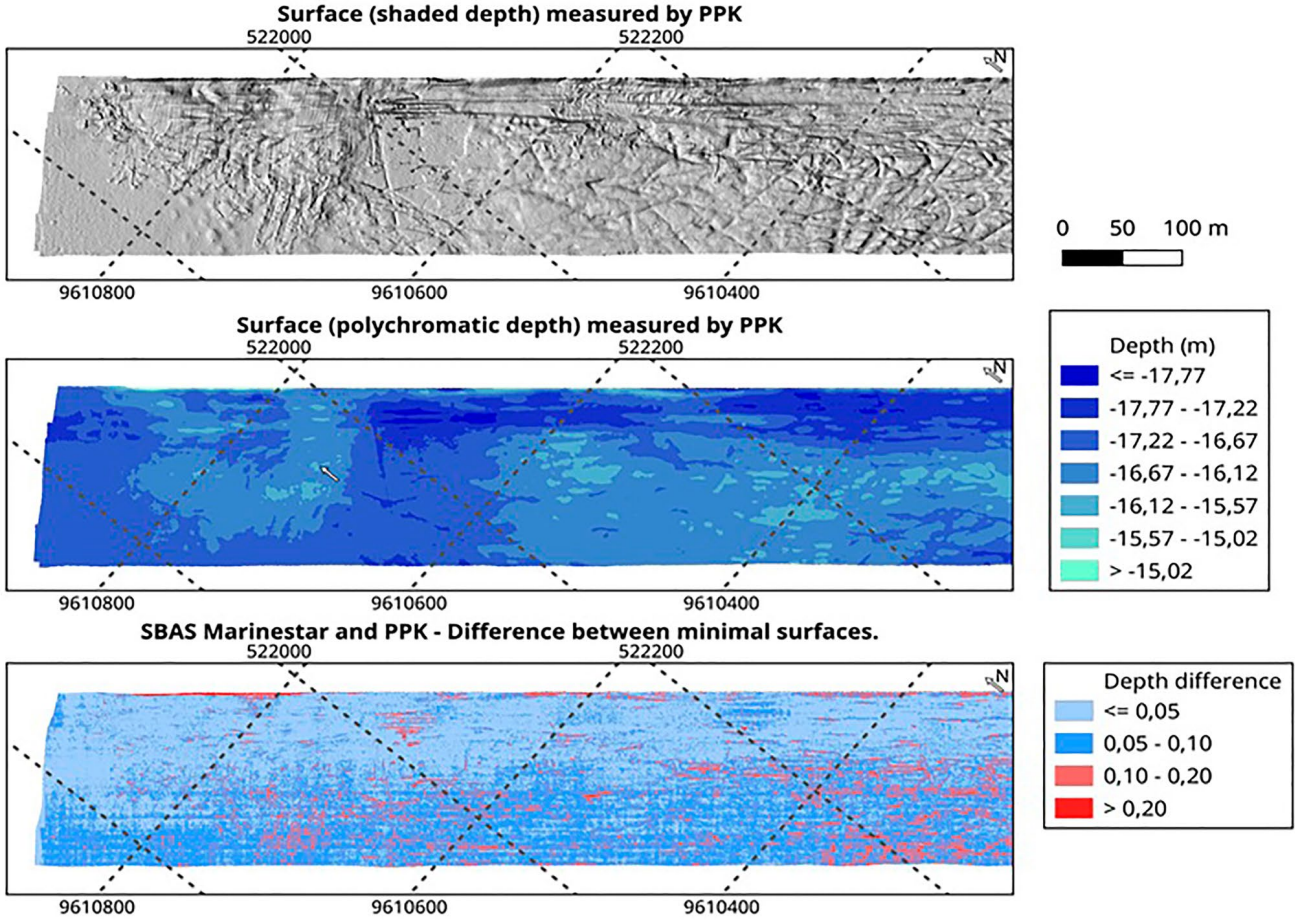
Comparison of models.

In Fig. 13, 3 visualization maps are presented. The third map shows the illustration of a model that represents the difference between the minimum surfaces resulting from each positioning method. The first two maps represent the hydrographic base obtained by method 2 (PPK), which was generated and used by the Brazilian Navy to update the nautical chart 710. From interpretation of the comparative map (third), it is possible to notice that the greatest differences continue to occur in areas with large discontinuities, easily observed by the roughness of the first map (shading).

To obtain a more careful comparison, a histogram was created to verify the difference between the two types of products that could be delivered to the Brazilian Navy.

It is worth mentioning that, thus far, professionals registered with the Directorate of Hydrography and Navigation use MarineStar due to the convenience of being an absolute means of screening and a method that has been used in some surveys since 32% of the surveys authorized by the Brazilian Navy were taken.

**Figure 14:**
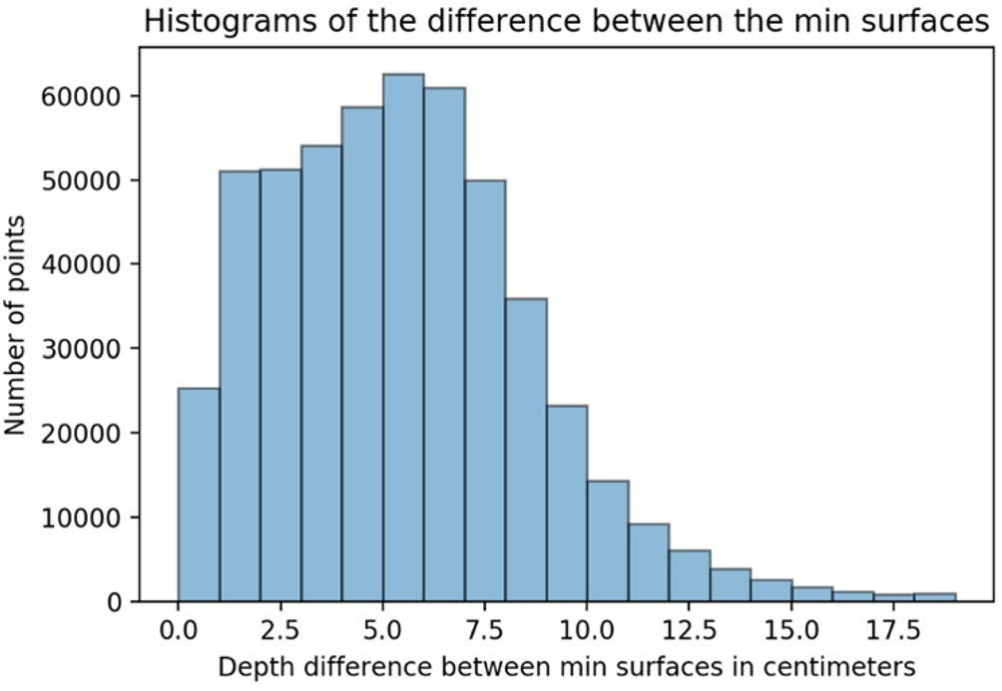
Histogram of the number of points by precision. Data extracted from the PosPac MMS program.

Figure 14 presents the histogram, which shows the number of points per range of depth differences. The largest number of points was between 5 and 6 cm. There were points with a difference of up to 19 cm.

From Fig. 14, it is possible to notice that the MarineStar has compatible accuracy for several applications and that it would perfectly suit the hydrographic survey to serve in engineering works that do not have as much cartographic rigor as the hydrographic survey of Exclusive Order, since it is not used for updating or manufacturing of new hydrographic charts.

## Conclusions

The comparative analysis was carried out over the entire area using all the points, identifying the common nodes of the two surfaces in a mesh generated at 0.5 m intervals. This comparison facilitated the identification of nodes outside the specified range compared to the entire surveyed region.

Method 2 (the PPK method) obtained better positional results for the vessel. The PPK coordinates contributed to better final vertical results in the hydrographic survey. The survey produced by Method 2 was sent and used without restriction by the Brazilian Navy for hydrographic surveys of the exclusive order. The results of the hydrographic survey were used to update the Nautical Chart 710, which encompasses the area of the Port of Pecém.

Depending on the behavior of the seabed, the vertical uncertainty may be more compromised depending on the positioning method applied. There may be a connection between depth irregularities in the elevation model and the need for more accurate vessel coordinates. The study showed that in the most homogeneous areas, the two methods were efficient and did not result in great disparities in the levels of difference for each positioning method.

The comparison between the two generated hydrographic bases illustrates that the vessel positioning method is a relevant factor for the success of the hydrographic survey, meeting the S-44 classification criteria. In this case, only the survey produced by Method 2 adequately met the requirements for updating operational parameters and consequently the cartographic update, thus contributing to the economic development of the country without degrading navigation safety.

It was found that the hydrographic survey developed using Method 1 is highly accurate and can be used in surveys where the technical rigor is not too high, such as the hydrographic survey of the exclusive order.

It became clear that it is possible to perform a hydrographic survey of the exclusive order using Method 1; however, there is a need to develop a more careful follow-up to increase the hydrographic survey coverage number (to saturate the area) to meet the demand of the Brazilian Navy for differences of up to 10 cm in 95% of the cases for each pair of depths obtained within the same cross-section.

According to the results of the comparative study carried out, for areas where irregularities are very severe, it is not convenient to use Method 1 since, in this case, better horizontal precision is essential to meet the precision recommendations for exclusive order.

The results indicate that it is possible to increase the percentage of use of hydrographic surveys of the exclusive order authorized by the Brazilian Navy using a more accurate GNSS method, such as kinematic postprocessing, or that at least it could be faster because there is no need for saturation of the entire survey area.

As a suggestion for the development of future studies and scientific collaboration, it is convenient that the same subject of concern be developed in other areas. It also follows the proposal to carry out a hydrographic survey of the exclusive order using Method 2 for areas less commonly used by the Brazilian Navy.

## Data Availability

The data produced with both methods is available at https://drive.google.com/drive/folders/1ahjbbpmFn0IxrbHeuigcLRGITtJxOhet?usp=sharing. The folder contains the 50 cm regular grids representing the difference between the minimum and maximum depth surfaces and the grid that represents the difference between the maximum depth surfaces produced with each method.
